# Enhancing Heavy Metal Detection through Electrochemical Polishing of Carbon Electrodes

**DOI:** 10.3390/bios14090412

**Published:** 2024-08-24

**Authors:** Sanjeev Billa, Rohit Boddu, Shabnam Siddiqui, Prabhu U. Arumugam

**Affiliations:** 1 Institute for Micromanufacturing (IfM), Louisiana Tech University, Ruston, LA 71272, USArbo012@latech.edu (R.B.); 2 Center for Biomedical Engineering and Rehabilitation Science (CBERS), Louisiana Tech University, Ruston, LA 71272, USA

**Keywords:** electrochemical, sensors, heavy metals, cadmium, lead, carbon, screen printing, electrocatalyst

## Abstract

Our research addresses the pressing need for environmental sensors capable of large-scale, on-site detection of a wide array of heavy metals with highly accurate sensor metrics. We present a novel approach using electrochemically polished (ECP) carbon screen-printed electrodes (cSPEs) for high-sensitivity detection of cadmium and lead. By applying a range of techniques, including scanning electron microscopy, energy-dispersive spectroscopy, Raman spectroscopy, electrochemical impedance spectroscopy, and cyclic voltammetry, we investigated the impact of the electrochemical potential scan range, scan rate, and the number of cycles on electrode response and its ability to detect cadmium and lead. Our findings reveal a 41 ± 1.2% increase in voltammogram currents and a 51 ± 1.6% decrease in potential separations (*n* = 3), indicating a significantly improved active electrode area and kinetics. The impedance model elucidates the microstructural and electrochemical property changes in the ECP-treated electrodes, showing an 88 ± 2% (*n* = 3) decrease in the charge transfer resistance, leading to enhanced electrode electrical conductivity. A bismuth-reduced graphene oxide nanocomposite-modified, ECP-treated electrode demonstrated a higher cadmium and lead sensitivity of up to 5 ± 0.1 μAppb^−1^cm^−2^ and 2.7 ± 0.1 μAppb^−1^cm^−2^ (*n* = 3), respectively, resulting in sub-ppb limits of detection in spiked deionized water samples. Our study underscores the potential of optimally ECP-activated electrodes as a foundation for designing ultrasensitive heavy metal sensors for a wide range of real-world heavy metal-contaminated waters.

## 1. Introduction

The rapid increase in industrialization and urbanization globally has resulted in a higher release rate of toxic chemicals, particularly heavy metals, into the environment and the human food chain. This poses significant risks to both human health and the ecosystems we depend on. Common heavy metals like cadmium, lead, arsenic, and mercury are released into the environment through industrial waste disposal, run-off, mining, and agricultural activities. Cadmium (Cd^2+^) and lead (Pb^2+^) are particularly prevalent in the environment due to their use in pipes, batteries, and the electroplating industry [1,2]. Human exposure to these heavy metals through inhalation, ingestion, and dermal absorption can affect vital organs such as the lungs, kidneys, the central nervous system, and the cardiovascular system, leading to a range of health issues, including hypertension, autoimmune diseases, renal failure, and osteoporosis [3,4,5,6]. There is no safe level of exposure to heavy metals, and even low levels can result in serious problems, especially for children and pregnant women [7,8,9,10]. Given the health risks associated with heavy metal exposure, monitoring these substances in the environment has become a crucial goal of research.

Environmental trace monitoring of heavy metals is generally performed using bulky, sophisticated, and expensive lab-based analytical techniques such as high-performance liquid chromatography, inductively coupled plasma mass spectrometry, atomic emission spectroscopy, atomic absorption spectroscopy, and laser-induced breakdown spectrometry [11,12,13,14,15,16,17]. Though these techniques can detect a large panel of elements with high sensitivity and extremely low detection limits (LODs), they suffer from several drawbacks [17]. They require pre-concentration and separation techniques and expensive reagents, rely on qualified technicians to perform the multi-step sample preparation, and require complex and expensive equipment. Less expensive do-it-yourself kits for multiple metal ions are available, but their reported lack of specificity and reliability are serious limitations [18]. Accurate and reproducible heavy metal testing is limited only to centralized laboratories, where water samples must be collected and transported for testing, which is time-consuming and not performed in real time.

Given the challenges mentioned above, electrochemical microsensors with electrocatalyst particle-modified carbon electrodes coupled with pulsed voltammetric detection methods, especially square wave anodic stripping voltammetry (SWASV), are commonly employed for detecting heavy metals [19,20]. Electrode materials must be carefully designed and prepared for the electrochemical detection of heavy metals with high sensitivity and selectivity and low LODs in the sub-parts per billion (ppb) range. Carbon materials for the working electrode are strongly preferred because their surface chemistry and mechanical and electrochemical properties provide facile redox reactions, positively influencing the sensing performance [21,22]. Electrochemical treatments such as ECP, electrochemical roughening, laser etching, and plasma etching are commonly used to activate carbon working electrodes in electroanalytical sensing applications. Generally, these treatments boost sensor metrics such as sensitivity, selectivity, response time, signal-to-noise ratio, LODs, and useful lifetime for various types of analyte detection. Such treatments are expected to improve the electrode performance by removing adventitious adsorbates from the electrode surface, changing the graphitization and orderliness of the carbon surface, and breaking the graphitic edge planes into smaller, rougher, more active microregions, presumably at defects, thereby increasing the edge plane defect density and changing the O/C ratio, the surface oxide states, oxide coverage, and the surface functional groups. These microstructural and surface chemistry changes were observed and investigated in this work.

These carefully surface-prepared carbon electrodes are further suitably modified with electrocatalysts as nanocomposites with bismuth (Bi), molybdenum disulfide (MoS_2_), chitosan, reduced graphene oxide (rGO), carbon nanotubes (CNTs) [23,24,25,26,27,28,29], etc., which deliver the necessary enhancements in sensitivity and selectivity for heavy metals, including Cd^2+^ and Pb^2+^. Being less toxic, Bi is the best electrocatalyst for creating more binding sites and can form fused alloys with heavy metals. While detecting Pb^2+^, Bi helps reduce Pb^2+^ to form a Pb-Bi alloy during pre-concentration. During the second step, the fused Pb-Bi alloy may readily oxidize as Pb^2+^ ions dissolve into the solution [26]. GO, a 2-D monolayered hexagonal lattice structured *sp*^2^ hybridized carbon is considered another potential electrode material in heavy metal detection because of its high electrical conductivity, heterogeneous electron transfer rates, large surface area, abundant surface functional groups, and additional transport paths for electrons and ions at a relatively low cost [21]. Considering these unique properties, for the first time, a Bi-rGO nanocomposite [30] with new and abundant heterojunctions was prepared and used in the surface modification of the ECP-treated cSPEs to detect heavy metals.

SWASV enables the simultaneous detection of multiple heavy metals using an electrode (sensor) array connected to a portable potentiostat. Each heavy metal ion has its potential scan range for anodic stripping (e.g., −0.9 to −0.7 V for Cd^2+^, −0.6 to −0.4 for Pb^2+^, 0 to +0.2 V for As^3+^, respectively) [22,30]; thus, the simultaneous presence of such metal ions should show no interference. SWASV utilizes a two-step method: preconcentration and then stripping/detection. During the preconcentration of the metal(s), heavy metal ions in the sample solution are reduced, concentrated, and deposited onto the working electrode surface at their respective standard electrode cathodic (negative) potentials. In the second step, the pre-concentrated heavy metals are stripped from the electrode and redissolved by scanning the potential from a negative to a positive voltage with a suitable frequency, amplitude, and step potential [26].

This study utilized a multi-array cSPE with eight individually electrically addressable electrodes. We aimed to systematically investigate the impact of ECP parameters such as the electrochemical potential scan range, scan rates, and the number of scan cycles on electrode properties and also the effect of applying an optimal Bi-rGO nanocomposite coating on the ECP-treated electrodes to determine their sensitivity for detecting Cd^2+^ and Pb^2+^. The hypothesis that is being proposed is that the ECP-treated cases should serve as a highly conductive surface with a high electroactive surface area and minimal interfacial electrical resistance. These near-optimal electrode properties effectively shuttle the detection currents from the nanocomposite layer to the transducer, resulting in higher sensitivities and lower LOD values.

## 2. Materials and Methods

### 2.1. Chemicals

The chemicals purchased from Sigma Aldrich (St. Louis, MO, USA) for this work included sulfuric acid (CAS. 7664-93-9), potassium chloride (CAS. 7447-40-7), potassium hexacyanoferrate (II) trihydrate (CAS. 14459-95-1), potassium hexacyanoferrate (III) (CAS. 13746-66-2), bismuth (III) nitrate pentahydrate (CAS No. 10035-06-0), graphene oxide powder, ethylene glycol (CAS No. 107-21-1), sodium borohydride (CAS No. 16940-66-2), dimethylformamide (CAS No. 68-12-2), sodium acetate (CAS No. 127-09-3), acetic acid (CAS No. 64-19-7), cadmium standard for AAS (Product No. 51994), and lead standard for AAS (Product No. 16595). A saturated calomel reference electrode (RE) was purchased from Gamry Instruments and was used for the ECP.

### 2.2. Morphological and Structural Characterization

The surface morphology and chemical composition of the cSPEs were examined using a field-emission scanning electron microscope (FESEM: Hitachi S-4800 (Urbana, IL, USA)). In addition, the films were characterized by Raman spectroscopy (Control Development 2DMPP with λ: 514 nm). Peak fitting was carried out using the Thermo Advantage Data system.

### 2.3. Electrochemical Characterization

The cSPE chip consists of eight individually electrically addressable working electrodes (WEs) (2.95 mm diameters), a built-in carbon ring as counter electrode (CE), and a Ag/AgCl reference electrode (RE) situated at the center which is equidistant from all the working electrodes (Supplementary Figure S1a). The WEs were first ECP-cleaned in 0.1 M H_2_SO_4_ by cycling at varying electrochemical potential scan ranges (±0.5, ±1.0, ±1.5, and ±2.0 V) with a fixed 20 mV/s scan rate for 10 cycles to identify the optimal potential scan range to activate the electrodes electrochemically. Next, the two other ECP parameters, scan rate and cycles (20 and 40 mV/s: 10, 20, and 30 cycles), were investigated using STAT-i-MULTI8 Multichannel potentiostats (Metrohm DropSens, Riverview, FL, USA). The ECP-treated electrodes were characterized for electrochemical behavior using cyclic voltammetry (CV) and electrochemical impedance spectroscopy (EIS). CV was performed at 100 mV/s using the 5 mM ferro/ferricyanide redox couple in a supporting electrolyte of 1 M KCl. Using the same solution, EIS was recorded between 100 kHz and 100 MHz with a 10 mV AC signal amplitude (rms value) at open circuit potential (OCP).

### 2.4. Nanocomposite Preparation and Surface Modification of cSPEs

For heavy metal detection, cSPE is modified with a (BiO)_2_CO_3_-rGO nanocomposite prepared as per reference [30]. A 100 mL solution mixture was prepared by adding 50 mL of DI water and 50 mL of ethylene glycol. Then, 500 mg each of Bi (NO_3_)_3_·5H_2_O and GO was added to the solution and stirred for 30 min at 300 rpm and 60 °C. The prepared mixture was then chemically reduced by adding 2 mL of a 0.51 g NaBH_4_ reducing agent drop-wise at 60 °C on a hot plate with continuous stirring at 300 rpm for 2 h. Then, the solution mixture was centrifuged for 15 min at 4500 rpm until the nanocomposite settled at the bottom. Then, the centrifuge step was repeated 3× in DI water (10 min each) to remove the solvent residues (Supplementary Figure S1b). The nanocomposite deposit thus obtained was transferred into a glass Petri dish and placed in a convection oven to dry completely at 50 °C overnight for 14 h. This yields about 1 g of nanocomposite. Finally, 1 mg of the nanocomposite is added to 4 mL DMF and sonicated for 20 min. Then, the resulting nanocomposite ink was drop-cast (~0.2 μL) onto the electrodes using a Hamilton microsyringe (Supplementary Figure S1c). The cSPE chip was then cured in the oven at 60 °C for 1 h and calibrated at varying Cd^2+^ and Pb^2+^ concentrations (up to 30 ppb).

### 2.5. SWASV Detection of Cd^2+^ and Pb^2+^

Calibration of Cd^2+^ and Pb^2+^ concentrations was carried out using (BiO)_2_CO_3_-rGO surface-modified electrodes in a 0.1 M acetate buffer first at varying pH (4.5 to 4.8) using a 3-electrode electrochemical setup and using a STAT-i-MULTI8 multichannel potentiostat (Metrohm DropSens). The heavy metals were deposited at −1.2 V for 200 s (pre-concentration step) onto the WEs. For detection (stripping step), a potential scan was applied from −1.25 V to −0.45 V with a frequency of 25 Hz, an amplitude of 25 mV, a step potential of 5 mV, and an equilibrium time of 10 s. The built-in Ag/AgCl and carbon ring electrodes were used as reference/counter electrodes.

## 3. Results and Discussion

### 3.1. Effect of ECP’s Potential Scan Range on Electrochemical Properties

**Figure 1:**
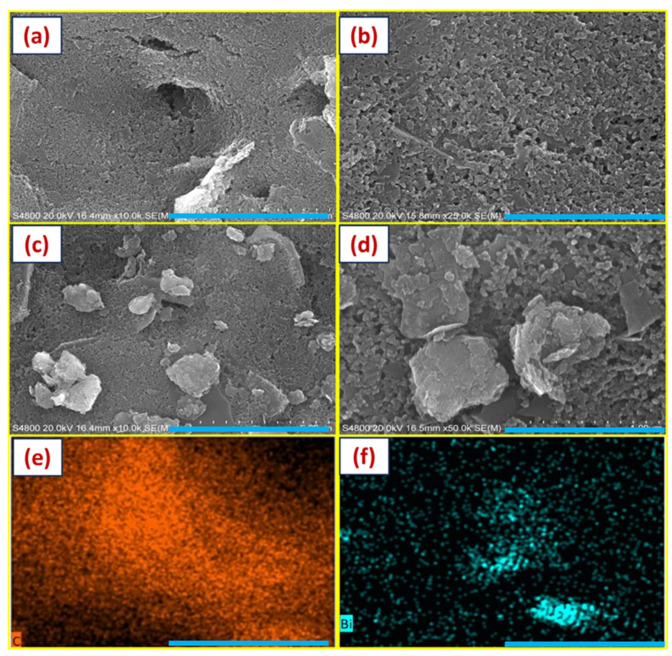
SEM and EDS images showing (**a**,**b**) un-modified cSPEs; (**c**,**d**) (BiO)_2_CO_3_-rGO nanocomposite-modified WE; (**e**,**f**) elemental mapping of un-modified and nanocomposite-modified WE. Scale bars for (**a**,**c**,**e**), (**b**), and (**d**,**f**) are 5 μm, 2 μm, and 1 μm, respectively.

**Figure 2:**
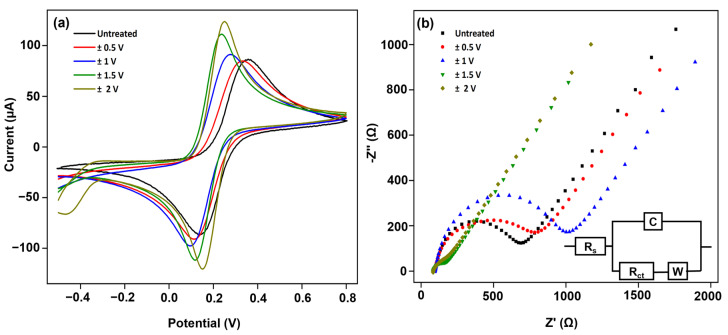
Effect of electrochemical potential scan range on cSPE properties. (**a**) Overlay of cyclic voltammograms recorded from the carbon SPE electrochemically cleaned at different potential scan ranges ±0.5 V (black), ±1 V (green), ±1.5 V (blue), ±2 V (orange) and with 20 mV/s; 10 cycles. The scan rate is 100 mV/s. (**b**) Overlay of Nyquist plots at 10 mV amplitude, 0.1 Hz–100 kHz. (Inset) Equivalent circuit model. The electrolyte is 5 mM Fe (CN)_6_^3−/4−^ in 1 M KCl.

The ECP process exposed the electrodes to electrochemical cycling at varying potential scan ranges, a fixed 20 mV/s scan rate, and 10 scan cycles in a 0.5 M H_2_SO_4_ electrolyte bath. The objective was to identify the optimal scan range to fully activate the carbon electrodes with the desirable surface defects that could significantly increase the surface electrical conductivity and decrease the charge transfer resistance [31]. Based on prior work, we chose the scan ranges to be ±0.5, ±1.0, ±1.5, and 2.0 V. For the first time, we studied the ECP treatment effect (i.e., potential scan range) on the electroactive area, charge transfer resistance, and Cd^2+^ and Pb^2+^ sensitivity. This work was conducted with the knowledge that a highly activated electrode surface is crucial before the electrocatalyst-embedded nanocomposite is coated to decrease the interfacial resistance and achieve a higher detection sensitivity. ECP-treated electrodes demonstrated a highly activated surface (Figure 1a,b) with no film delamination. Figure 2 shows the electrochemical CV and EIS responses in the ferro/ferricyanide redox couple. Table 1 shows the effect of the potential scan range on the key electrochemical characteristics. Electrode reaction kinetics data can be obtained from the peak potential separations (Δ*E_p_*) between the forward and reverse peak currents (E*_anodic_*−E*_cathodic_*) of the redox system. Studies show Δ*E_p_* and the associated slope of the cyclic voltammogram from inner- and outer-sphere redox systems could be a reliable CV indicator for studying electrode reaction rates. From the voltammograms, the forward oxidation peak currents (*i_pf_*) increased for all the potential scan ranges, which directly measures the electroactive area. A maximum increase of 23 ± 1.4% (*n* = 3) was observed in the ±2.0 V potential scan range. Also, in the same potential scan range, the Δ*E_p_* decrease, an indicator of electrode kinetics, was the highest at −38 ± 1.5% (*n* = 3). The lowest Δ*E_p_* value of 90 mV after the ECP treatment in a ±2.0 V potential scan range (150 mV for the untreated electrodes) is still larger than the value corresponding to the theoretical value of 59 mV for FeCN_6_^3−/4−^ (a 1e^−^ fully reversible redox reaction). This increase (or deviation) is expected from cSPEs that are contaminated and/or oxygen-terminated. However, no significant change was noted, and the Δ*E_p_* values were about 200 mV in the typical potential scan ranges (i.e., ±0.5 V and ±1.0 V) employed in the literature.

To understand how changes in the microstructure of treated electrodes affect the sensitivity of detecting Cd^2+^ and Pb^2+^, we conducted a thorough analysis using electrochemical impedance spectroscopy (EIS) and developed circuit models. This allowed us to identify specific regions on the electrode with distinct electrochemical activity, which has been reported in our previous work and the work of others [32,33,34,35,36,37]. EIS is widely used to investigate the electrochemical properties of materials, electrode processes, and interfaces [38,39]. The EIS Nyquist plot and the data fitting to Randle’s equivalent circuit are shown in Figure 2b. Each plot consists of an arc followed by a linear line. The arc represents the charge transfer resistance. It is evident from the figure that the arc of all the electrodes decreases after the ECP treatment, indicating a reduction in impedance post-treatment. The circuit includes elements such as *R_s_* (the high-frequency resistance of the electrolyte), *R_ct_* (the charge transfer resistance associated with ion injection from the electrolyte to the electrode surface), *W* (Warburg diffusion impedance due to the diffusion of the electroactive species from regions of high concentration to regions of low concentration), and *Y_0_* (a constant phase element (CPE) associated with the heterogeneity of the double-layer distributed capacitance on the electrode surface, also known as capacitive dispersion or *C* (capacitance)). The CPE provides information regarding surface roughness heterogeneity and the conductivity of a double layer of the electrode. The surface roughness and heterogeneity information are gained through the ‘N’ value, and conductivity or admittance information is gained through the *Q* value. The *W* element provides information about the diffusional impedance, and the charge transfer resistance provides information about the interfacial impedance of the electrode-electrolyte. The circuit illustrates that the current follows two parallel pathways: the CPE-*R_ct_* and the Warburg element.

For the ±0.5 V and ±1.0 V treatment scan ranges, there is no significant decrease in *R_ct_* for the electrodes; rather, there is an increase to ~750 Ω. This indicates that the overall resistance to electron transfer between the electrode-electrolyte interfaces does not decrease after the ECP treatment. Furthermore, the CPE decreases slightly for the ±0.5 V treated electrodes and increases for the ±1.0 V treated electrodes, suggesting that the impedance of the electrode surfaces remains the same and/or suffers a slight decrease. Additionally, the Warburg element shows minimal changes for all the treated electrodes. The data suggest that the ECP treatment with smaller potential scan ranges is ineffective in significantly improving electrode conductivity. For the ±1.5 V treated electrodes, the *Y*_0_ is replaced by *C*. The fitting of the C element instead of the CPE element after the ECP treatment demonstrates that the electrode surface has become smoother and more homogenous due to uniform current density distribution experienced at the electrode surface. The ΔY_0_ represents the electrode’s surface impedance with an N value less than 1 for inhomogeneous surfaces. A positive value of ΔY_0_ indicates a decrease in the surface impedance and an increase in the surface conductivity and vice versa. The ΔC represents the electrode’s capacitive impedance with an N value of 1 for smooth surfaces. A positive value of ΔC indicates a decrease in the surface impedance and an increase in the surface conductivity and vice versa. The *R_ct_* decreases ~7 times to ~80 Ω, suggesting a significant improvement in electrode conductivity. Further, the electrode surface conductivity increases as the capacitive impedance of the electrode surface reduces by 5 times, which is inversely proportional to *C*. Further, Warburg impedance also decreases slightly. For the ±2.0 V treated electrodes, the *R_ct_* decreases ~9 times to ~56 Ω, and the capacitive impedance reduces ~11 times, resulting in an 88 ± 2% (*n* = 3) decrease in the *R_ct_*, suggesting significant enhancements in the surface electrical conductivity. The Warburg impedance also decreases slightly. This suggests that ±2.0 V is the optimal ECP potential scan range, which removed the surface adsorbates and activated the carbon electrode surface. The experimental data and observations consistently indicate a notable increase in the electroactive area and electrode kinetics by the ECP treatment process.

### 3.2. Effect of ECP’s Scan Rate and Number of Cycles on Electrochemical Properties

**Figure 3:**
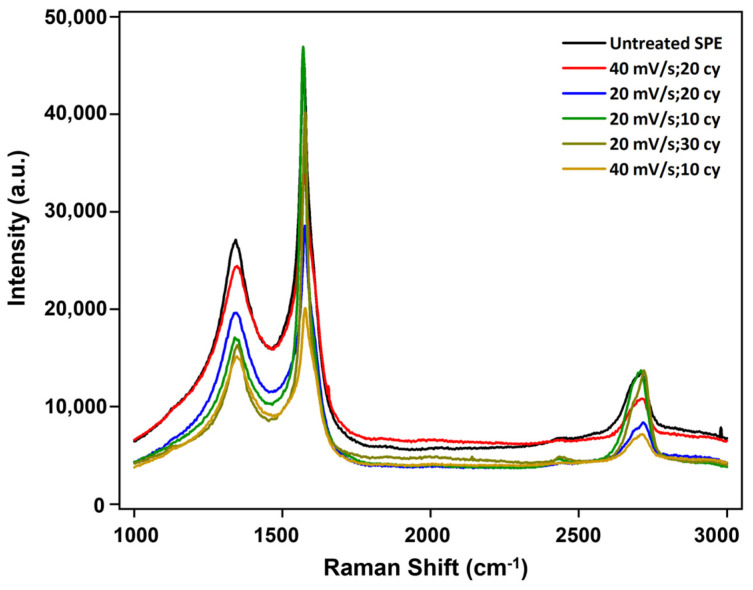
Raman spectra of ECC-treated cSPEs. The effect of ECC parameters, ±2.0 V scan range, with scan rates and number of scan cycles (20 mV/s: 10, 20, 30 cycles—green, blue, dark yellow curves; 40 mV/s: 10, 20 cycles—gold and red curves; and the untreated cSPE represented in black) on surface defect types and densities.

Next, we investigated the effect of the scan rate (20, 40 mV/s) and the number of scan cycles (10, 20, 30 cycles) on the electrode properties by fixing the potential scan range at ±2.0 V. The main objective is to identify an optimal condition to remove the surface adsorbates and activate the carbon’s edge planes and defects for maximum electron exchange between the electrode and the analyte. The Raman spectra of the untreated and ECP-treated electrodes are shown in Figure 3. We examined the changes in the intensity of the *D* and *G* bands in the spectra. The *D* band, observed at 1347 cm^−1^, signifies disordered graphite, indicating distortions in the *sp*^2^ crystalline graphite structure. Conversely, the *G* band, appearing at 1570 cm^−1^, reflects graphene behavior, attributed to the *E2g* mode arising from in-plane vibrations of *sp*^2^ carbon atoms arranged in a hexagonal lattice structure. Data analysis (Table S1) reveals that the largest increase in the D/G intensity ratio (~28%) occurs at the highest scan rate and the smallest number of cycles (40 mV/s, 10 cycles), suggesting increased structural disorderliness with more defects. Furthermore, a broader full width half maximum (FWHM) value indicates a greater structural heterogeneity or disorder. The FWHM decreases as both scan rate and the number of cycles increase, except for the 40 mV/s and 20 cycle conditions, indicating that higher scan rates cause the surface to be more orderly, which is consistent with the increase in the *D*/*G* intensity ratio [40,41,42].

**Figure 4:**
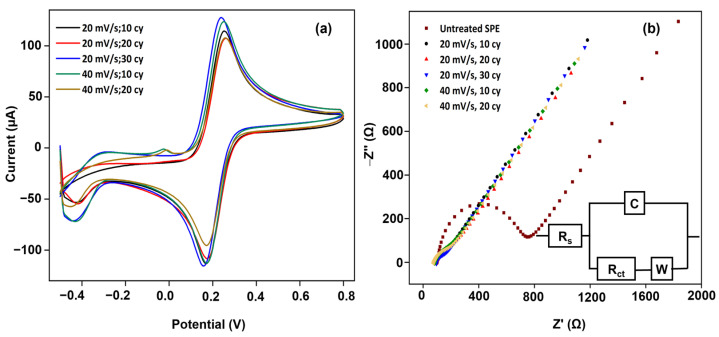
(**a**). Typical cyclic voltammograms were overlayed from the cSPEs ECP-treated at different scan rates and with a different number of cycles (black curve—uncleaned. 20 mV/s: 10, 20, 30 cycles—blue, light green and orange curves. 40 mV/s: 10, 20 cycles—magenta and dark green curves). The electrolyte is a 5 mM Fe(CN)_6_^3−/4−^ in 1 M KCl solution. Scan rate is 100 mV/s. (**b**) Representative Nyquist plots of screen-printed carbon electrodes. Typical EIS spectra of cSPE before (black dotted) and after (red dotted) ECP treatment at 40 mV/s and 10 cycles. The electrolyte is 5 mM Fe(CN)_6_^3−/4−^ in 1 M KCl, with a 10 mV amplitude of 0.1 Hz–100 kHz.

Figure 4a shows the overlay cyclic voltammograms of the cSPEs that are ECP-treated at 20 mV/s (10, 20, 30 cycles) and 40 mV/s (10, 20 cycles). The ECP-treated electrodes generally showed an increase in the *i_pf_* and a decrease in the Δ*E_p_*. The treatment has a more pronounced effect at either lower scan rates and a higher number of cycles or higher scan rates and a lower number of cycles. This resulted in an *i_pf_* increase of up to 41 ± 1.2% and an Δ*E_p_* decrease of up to −51 ± 1.6% (*n* = 3). Figure 4b shows the Nyquist plots and the data fitting to the equivalent circuit. Table 2 shows the effect of the scan rate and number of cycles on the key fitted circuit elements. Again, fitting the *C* element instead of the CPE element after the ECP treatment demonstrates that the electrode surface has become smoother and more homogenous due to uniform current density distribution experienced at the electrode surface. The *R_ct_* has decreased by several orders of magnitude due to increased exchange current. Since the exchange current is inversely proportional to the active area of the electrode, this implies that the active area of the electrode has increased by several orders of magnitude with the removal of the adventitious adsorbates from the electrode surface and breaking of the graphitic edge planes into rougher, more active microregions, presumably at defects. Due to increased exchange current, the *R_ct_* has decreased from approximately 480 Ω to 50 Ω. Further, there is also incremental improvement in the value of the *W* element. This further implies that the diffusional impedance decreased after the ECP treatment. All the electrodes treated with more cycles show a decrease in capacitive impedance. This suggests that a higher number of scan cycles not only causes the smoothing of the surfaces but also increases their conductivities. Further, increasing the scan rate does not significantly increase the *R_ct_*. The ECP treatment has increased the microstructural heterogeneity (e.g., shallow pores and edge planes breakage/defects) on carbon surfaces, increasing the surface’s conductivity. This is mainly because the charge transfer resistance, responsible for electron transfer between the electrode and the analyte, inversely depends on the electrode surface area. Such microstructurally created defects on the electrode surface increase the electroactive area. This reduces the charge transfer resistance, increasing the electrode surface’s conductivity. These enhancements in the electrode conductivity are expected to decrease the interfacial electrical resistance between the cases and the nanocomposite coating, demonstrating higher detection sensitivity and lower LOD values.

### 3.3. Effect of pH on SWASV Detection of Cd^2+^ and Pb^2+^

**Figure 5:**
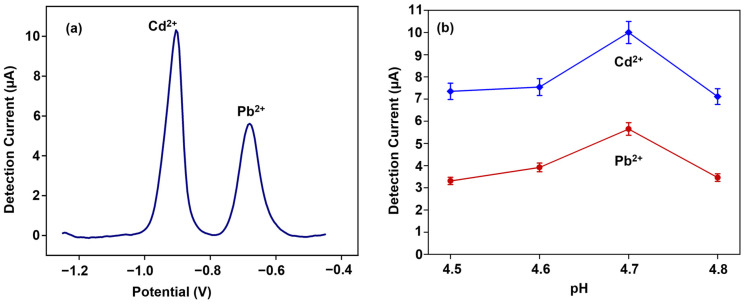
Effect of pH on detection currents. (**a**) A typical SWASV voltammogram of nanocomposite-modified cSPE. A 30 ppb Cd^2+^ and Pb^2+^ in 0.1 M acetate buffer, 4.7 pH, using DI water. The electrodes were ECP-treated at 40 mV/s, 10 cycles. For SWASV, the conditioning potential is 0.05 V; the deposition time is 200 s; the depositional potential is −1.2 V; the step potential is 5 mV; the amplitude is 25 mV; the frequency is 25 Hz; and the potential scan range is −1.25 V to −0.45 V. (**b**) Calibration data at different pH values (*n* = 3). Each data point averages 3 measurements from 3 WEs. The error bars represent ±1 standard deviation.

To achieve the desirable sensor metrics, we investigated the effect of pH on the detection sensitivity. Figure 1c–f shows the surface morphology and the elemental analysis of (BiO)_2_CO_3_-rGO modified cSPEs. The drop-casted nanocomposite cured at 60 °C for 1 h showed a more uniform loading and distribution across the electrodes. The presence of rGO helped achieve the high specific electrode area, and the presence of Bi particles helped in adsorbing the heavy metals during the pre-concentration step onto the electrode, even at very low concentrations (in ppb range). The pH affects the solution phase metal ions, which are affected by the presence and quantity of hydroxyl ions. A higher pH increases the hydroxyl ions, reducing the solution phase metal ions by forming metal hydroxide complexes. A lower pH reduces the solution phase metals, leading to the minimum availability of metal ions for deposition onto the electrode surface during pre-concentration. However, at pH levels that are too low, the protons compete with the heavy metal ions for the binding sites in the presence of the electrocatalysts on the surface-modified WE, affecting the sensor’s linear range and reproducibility [43]. Figure 5 shows the SWASV voltammograms of Cd^2+^ and Pb^2+^ at varying pH values, with 4.7 pH being the optimal value for achieving a higher detection sensitivity. The Cd^2+^ and Pb^2+^ peaks were detected at −0.9 V and −0.68 V vs. the Ag/AgCl reference electrode and exhibited high selectivity with sharp peaks and peak separations greater than 200 mV.

### 3.4. Effect of ECP Treatment on Cd^2+^ and Pb^2+^ Detection Sensitivity

**Figure 6:**
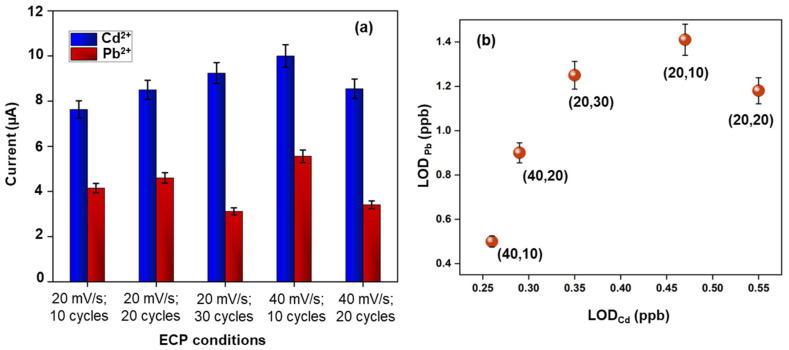
Calibration studies of 30 ppb Cd^2+^ and Pb^2+^ in 0.1 M acetate buffer at 4.7 pH, using DI water for different ECP conditions. Conditioning potential: 0.05 V; deposition time: 200 s; depositional potential: −1.2 V; step potential: 5 mV; amplitude: 25 mV; frequency: 25 Hz; potential scan range: +1.25 V to −0.45 V. (**a**) The calibration data for Cd^2+^ and Pb^2+^. Each data point is an average of 3 measurements from 3 electrodes. The error bars represent ±1 standard deviation. (**b**) LODs of Cd^2+^ and Pb^2+^.

**Figure 7:**
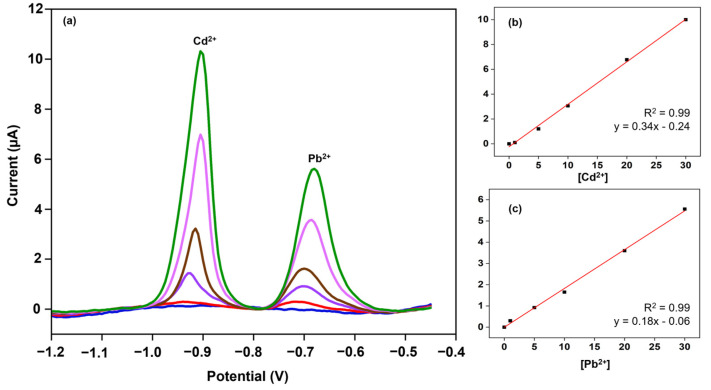
(**a**) SWASV voltammograms of Cd^2+^ and Pb^2+^ spiked at varying concentrations (0, 1, 5, 10, 20, and 30 ppb—blue, red, purple, brown, magenta, and green curves, respectively) in 0.1 M acetate buffer at 4.7 pH, using DI water. (**b**,**c**) Calibration curves of Cd^2+^ and Pb^2+^. Each data point averages three measurements from 3 WEs. The error bars represent ±1 standard deviation.

**Figure 8:**
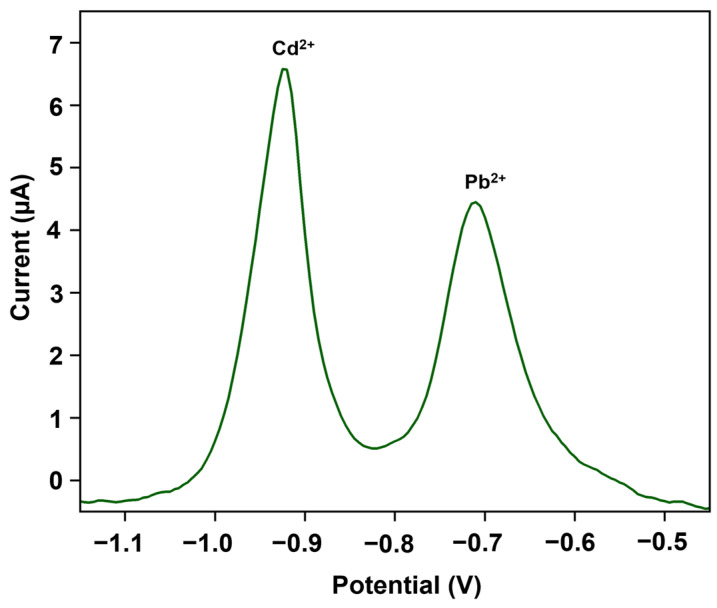
SWASV voltammogram of 30 ppb of Cd^2+^ and Pb^2+^ each in 0.1 M acetate buffer at 4.7 pH prepared in local tap water.

To study the effect of ECP on the detection sensitivity, we employed five different treatment conditions (20 and 40 mV/s; 10, 20, and 30 cycles) on the nanocomposite-modified cases. Figure 6a shows the detection currents at ECP-treated electrodes for 30 ppb spiked Cd^2+^ and Pb^2+^ samples prepared in 0.1 M acetate buffer at 4.7 pH. A 10% increase in the currents was observed for Cd^2+^ among the 20 mV/s treated electrodes at varying numbers of cycles. The largest detection current for Cd^2+^ was achieved at 40 mV/s and 10 cycles of treatment conditions. A similar trend was observed for Pb^2+^, with a 17% and 24% increase in the currents for the 20 mV/s treated electrodes with 10 and 20 cycles, respectively, and the largest current was observed at 40 mV/s and 10 cycles. By applying a treatment condition of 40 mV/s and 10 cycles, we performed the calibration studies (Figure 7) in a 0.1 M acetate buffer 4.7 pH ranging from 0 to 30 ppb (0, 1, 5, 10, 20, and 30 ppb) and the detection currents were comparable to the literature [30,44]. The voltammogram curves exhibited a monotonic linear relationship with increasing heavy metal concentrations. The magnitude of the peak currents can also be calculated using the linear regression equation shown in Figure 7b,c. The Cd^2+^ and Pb^2+^ sensitivities were 5 ± 0.1 μAppb^−1^cm^−2^ and 2.7 ± 0.1 μAppb^−1^cm^−2^ (*n* = 3), respectively, which are better than the ones reported in the literature [30,44,45,46], in terms of not employing toxic substances like mercury or additional permselective coatings (e.g., Nafion) or lengthy processing steps. Figure 6b shows the LOD values for the two heavy metals on different ECP-treated cSPEs. The LODs are calculated using 3 × (Standard Deviation/Slope). The data suggest the significance of ECP treatment and the choice of ECP parameters for achieving sub-ppb detection levels. Supplementary Figure S2 demonstrates the effect of ECP treatment on the detection sensitivities. The ECP treatment showed a 3-fold increase in Cd^2+^ sensitivity and a 2-fold increase in Pb^2+^ sensitivity. To validate the nanocomposite-modified electrodes’ “sensors”, we tested the initial prototypes using real-world tap water samples. The sensors acquired clear signals when spiked with 30 ppb of Cd^2+^ and Pb^2+^, the typical concentrations expected in real-world water samples (Figure 8).

We performed interference studies with two commonly found heavy metals, Zn^2+^ and Cu^2+^, at a ratio of 1:10 to demonstrate the sensors’ selectivity. The concentrations of Cd^2+^ and Pb^2+^ were 30 ppb each, and the concentrations of the Zn^2+^ and Cu^2+^ were 300 ppb each (Supplementary Figure S3a–c). The cSPEs were ECP-treated using 40 mV/s and 10 cycles and coated with the nanocomposite. During these studies, we further investigated the effect of pH and SWASV deposition potentials on heavy metal-specific detection selectivity. The data suggest that Cd^2+^ has an optimal deposition potential of −1.2 V and a pH value of 5. In the presence of Zn^2+^ as an interferent, a 4 ± 0.5% current drop was observed; in the presence of Cu^2+^, a 35 ± 2% current drop was observed (*n* = 3). Similarly, the data suggest that Zn^2+^ has an optimal deposition potential of −0.8 V and a pH value of 5. In the presence of Zn^2+^, an 18 ± 1% current drop was observed; in the presence of Cu^2+^, a 17 ± 1.5% current drop was observed (*n* = 3). We also observed a shift in the peak potentials in the presence of the interferents—further optimization of the coatings and identification of the heavy metal-specific SWASV parameters.

## 4. Conclusions

We have shown that ECP-treated cSPEs provide an excellent combination of key sensor metrics such as sensitivity, selectivity, and detection limit for two key heavy metals, Cd^2+^ and Pb^2+^. The complementary SEM, Raman, EDAX, and EIS spectra have demonstrated the ability to enhance the electrochemical activity from ECP-treated carbon electrodes, which can be tailored to improve further the electrochemical sensing performance of many heavy metals besides Cd^2+^ and Pb^2+^. For instance, by choosing an appropriate set of ECP process parameters and nanocomposite coating properties, the interfacial electrical properties can be customized to enhance the detection performance metrics. The ECP treatment increased the electroactive area and altered the carbon surface chemistry, which increased the overall electrical conductivity, electrode kinetics, reactivity, and selectivity. This work demonstrates that the properties of this new class of ECP-treated (BiO)_2_CO_3_-rGO nanocomposite-modified cSPEs depend on the nanocomposite composition, underlying carbon electrode microstructure, and surface functionalities. The key benefits of the proposed sensor are as follows: First, remarkable improvements in Cd^2+^ and Pb^2+^ sensitivity (5 ± 0.1 μAppb^−1^cm^−2^; 2.7 ± 0.1 μA ppb^−1^cm^−2^) and LOD (0.26 ppb; 0.5 ppb) offer great promise for advancing the field of environmental monitoring of heavy metals. Second, electrode activation can be selectively applied with a simple, scalable, low-cost ECP process for multiplexed heavy metal sensing. Third, this work will establish a new generation of ultrasensitive sensor arrays for real-time heavy metal detection studies.

## Figures and Tables

**Table 1:** CV and EIS data from the different ECC-treated cSPEs. A 5 mM ferro/ferricyanide redox couple was used. The scan rate is 100 mV/s (*n* = 3). For EIS, the % errors for R_s_, Y_0_/C, R_ct_, and W are 8–11%, 5–6%, 14–18%, 6–10%, 4–9%, and 3–5%, respectively.

ECP Potential Window (V)	Δ(Δi_pf_) (%)	Δ(ΔE_p_) (%)	ΔR_ct_ (%)	Δ(Y_0_) (%)	ΔC (%)
−0.5 V to +0.5	7 ± 0.1	−3 ± 0.1	+13 ± 2	−24 ± 5	—
−1 V to +1	6 ± 0.4	−3 ± 0.1	+51 ± 4	+58 ± 7	—
−1.5 V to +1.5	22 ± 1.4	−32 ± 1.6	−85 ± 3	—	+350 ± 11
−2 V to +2	23 ± 1.4	−38 ± 1.5	−88 ± 2	—	+ 945 ± 18

**Table 2:** CV and EIS data from the different ECC-treated cSPEs. A 5 mM ferro/ferricyanide redox couple was used. The scan rate is 100 mV/s (*n* = 3). For EIS, the % errors for R_s_, Y_0_/C, R_ct_, and W are 5–9%, 8–10%, 12–17%, 8–12%, 5–12%, and 3–8%, respectively.

ECP Condition	Δ(Δi_pf_) (%)	Δ(ΔE_p_) (%)	ΔR_ct_	ΔC	ΔW
20 mV/s; 10 cycles	23 ± 1.5	−38 ± 1.4	~7.5-fold decrease	~6.0-fold increase	~15.0-fold increase
20 mV/s; 20 cycles	24 ± 0.5	−38 ± 1.9	~10.0-fold decrease	~15.5-fold increase	~19.0-fold increase
20 mV/s; 30 cycles	35 ± 1.2	−48 ± 1.6	~10.0-fold decrease	~15.5-fold increase	~19.0-fold increase
40 mV/s; 10 cycles	41 ± 1.2	−42 ± 2.0	~8.5-fold decrease	~2.0-fold increase	~9.5-fold increase
40 mV/s; 20 cycles	23 ± 0.6	−36 ± 3.0	~8.5-fold decrease	~11.0-fold increase	~12.0-fold increase

## Data Availability

The original contributions presented in the study are included in the article/Supplementary Material; further inquiries can be directed to the corresponding author.

## Supplementary Materials

Applicable. The following supporting information can be downloaded at: https://www.mdpi.com/article/10.3390/bios14090412/s1, Figure S1: (a) cSPE chip, (b) (BiO)_2_CO_3_-rGO nanocomposite ink and (c) nanocomposite-modified carbon WEs. Table S1: Effect of ECP treatment on the D/G intensity ratio and the FWHM ratio. Figure S2: The effect of ECP treatment on Cd^2+^ and Pb^2+^ sensitivities. Figure S3: Selectivity studies.
