# Supplementary material for: Enhancing Heavy Metal Detection through Electrochemical Polishing of Carbon Electrodes
Source: Biosensors (Basel). 2024 Aug 24;14(9):412. doi: 10.3390/bios14090412 (PMC11429634; doi:10.3390/bios14090412)
Supplement: Supplementary file 1 [file biosensors-14-00412-s001.zip › biosensors-3064507-supplementary.pdf]

Supporting Information for

Enhancing Heavy Metal Detection through Electrochemical Polishing of Carbon Electrodes

Sanjeev Billa [1], Rohit Boddu[1], Shabnam Siddiqui[2], Prabhu U. Arumugam[1,2,]*

[1]Institute for Micromanufacturing (IfM), Louisiana Tech University, Ruston, LA 71272

[2]Center for Biomedical Engineering and Rehabilitation Science (CBERS), Louisiana Tech University, Ruston, LA 71272

[*]Corresponding author. Email: parumug@latech.edu

Postal address: 911 Hergot Ave, Institute for Micromanufacturing, Louisiana Tech University, Ruston LA 71272

Supplementary Figure S1

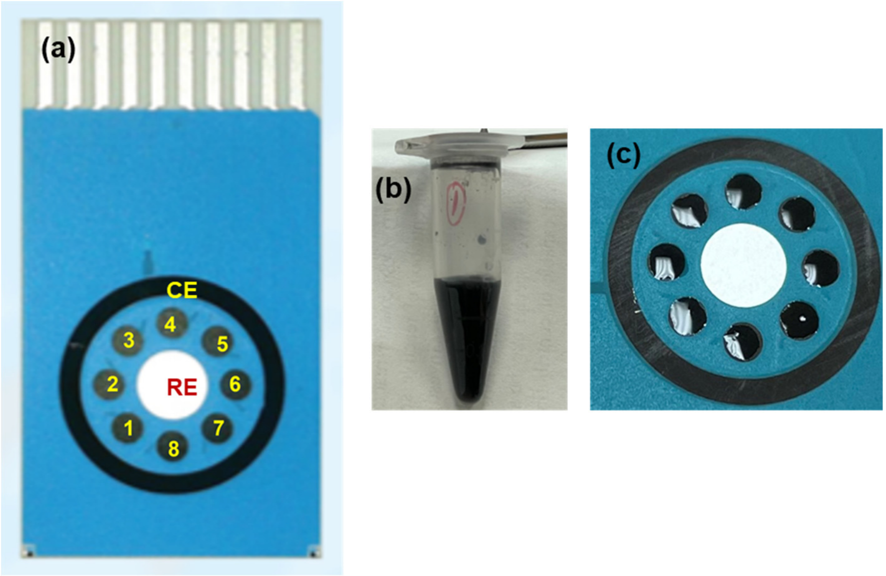

Figure S1. (a) cSPE chip with 8 WEs, built-in Ag/AgCl disk reference, and carbon ring counter electrodes. (b) $(BiO)_2CO_3$-rGO nanocomposite ink and (c) nanocomposite-modified carbon WEs.

Table S1. Effect of ECP treatment on the D/G intensity ratio and the FWHM ratio.

**ECP conditions**	I_D	I_G	I_D/I_G	$FWHM_D$	$FWHM_G$
No ECP	27190	45596	0.59	74.69	46.38
20 mV/s;10 cycles	17104	46931	0.36	64.90	33.70
20 mV/s;20 cycles	19655	28394	0.69	72.90	43.67
20 mV/s;30 cycles	16313	40225	0.41	50.23	27.53
40 mV/s;10 cycles	15179	20160	0.76	66.66	46.16
40 mV/s;20 cycles	24301	34182	0.71	79.77	51.84

Supplementary Figure S2

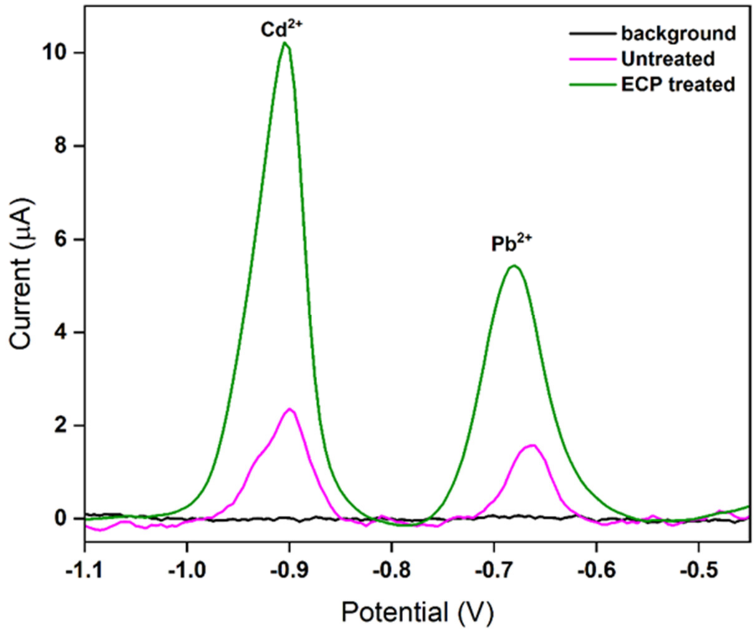

Figure S2. The effect of ECP treatment on Cd^{2+} and Pb^{2+} sensitivities. SWASV voltammogram of 30 ppb of Cd^{2+} and Pb^{2+} each in 0.1 M acetate buffer at 4.7 pH prepared in spiked DI water.

Supplementary Figure S3

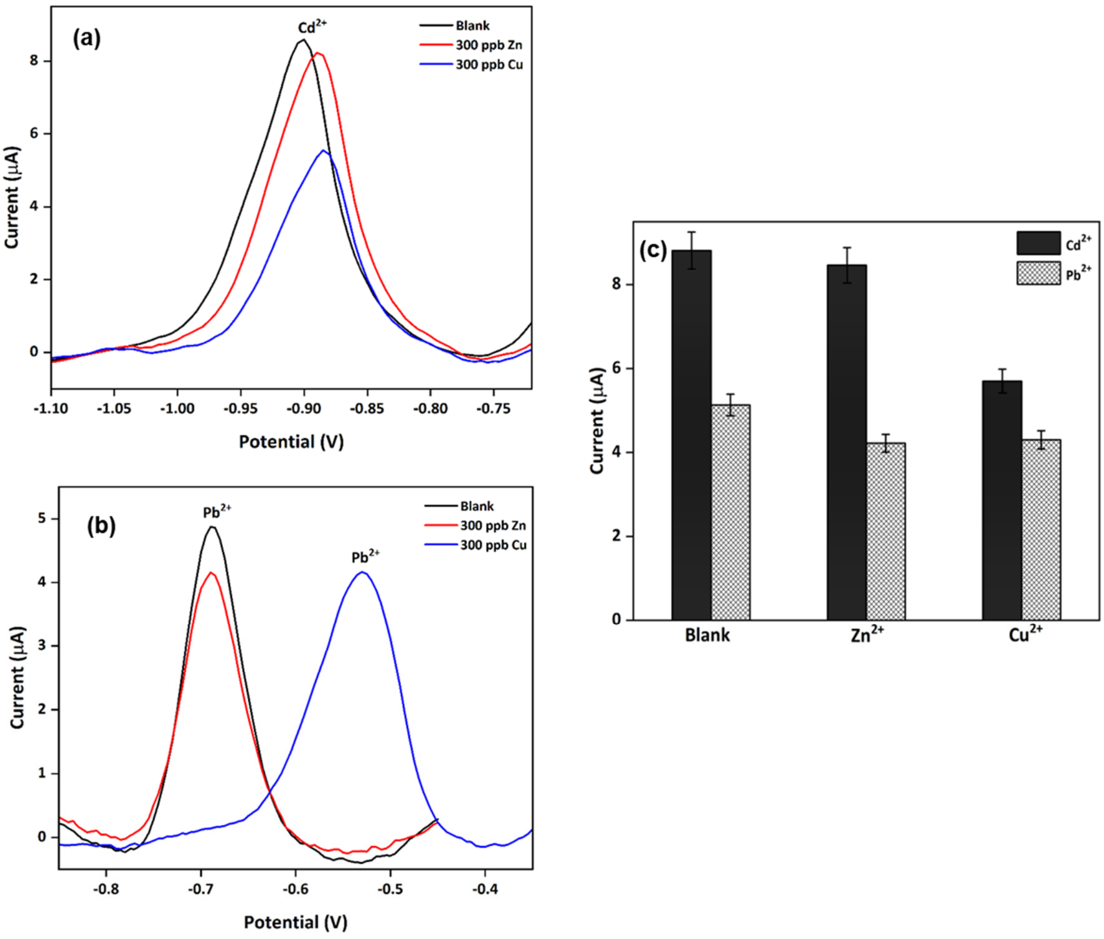

Figure S3. Selectivity studies. The SWASV voltammogram shows 30 ppb of Cd^{2+} and Pb^{2+}, and the concentrations of the Zn^{2+} and Cu^{2+} are 300 ppb each. The cSPEs were ECP treated using 40 mV/s and 10 cycles and coated with the nanocomposite. Used 0.1 M acetate buffer at 5.0 pH.
